# An In Vitro Study on the Efficacy of Mycotoxin Sequestering Agents for Aflatoxin B1, Deoxynivalenol, and Zearalenone

**DOI:** 10.3390/ani12030333

**Published:** 2022-01-29

**Authors:** Jong Young Ahn, Jongkeon Kim, Da Hyeon Cheong, Hyosun Hong, Jin Young Jeong, Beob Gyun Kim

**Affiliations:** 1 Department of Animal Science and Technology, Konkuk University, Seoul 05029, Korea; ahnjongyoung@gmail.com (J.Y.A.); gun5233@konkuk.ac.kr (J.K.); treasure8426@naver.com (D.H.C.); name_hyosun@naver.com (H.H.); 2 Animal Nutritional Physiology Team, National Institute of Animal Science, Rural Development Administration, Wanju 55365, Korea; jeong73@korea.kr

**Keywords:** aflatoxin B1, deoxynivalenol, mycotoxin sequestering agents, pigs, zearalenone

## Abstract

**Simple Summary:**

Mycotoxins in feeds can cause detrimental effects on the growth performance and health of pigs. One of the methods used to overcome the negative effects of mycotoxins in animal feeds is to add toxin sequestering agents to feed. The present work was conducted to determine the efficacy of mycotoxin sequestering agents using an in vitro method. All mycotoxin sequestering agents effectively bound to aflatoxin B1; only activated charcoal effectively sequestered deoxynivalenol; and a bentonite product, an aluminosilicate product, and activated charcoal effectively sequestered zearalenone.

**Abstract:**

The objective of this study was to determine the efficacy of mycotoxin sequestering agents for aflatoxin B1 (AFB1), deoxynivalenol (DON), and zearalenone (ZEA) using an in vitro method. The twelve toxin sequestering agents tested were seven bentonite products (bentonite A, B, C, D, E, F, and G), two aluminosilicate products (aluminosilicate A and B), a heulandite product, an activated charcoal product, and a yeast cell wall product. A two-step in vitro procedure was employed to mimic the conditions of temperature, pH, and digestive enzymes in the stomach and small intestine of pigs. All mycotoxin sequestering agents tested were able to bind to AFB1 with a high efficacy (>92%). The DON sequestering rate of activated charcoal (99.1%) was greater (*p* < 0.05) than that of other products. The ZEA sequestering rate of bentonite F (97.0%), aluminosilicate A (99.6%), and activated charcoal (100.0%) was the greatest (*p* < 0.05) among the tested mycotoxin sequestering agents. Overall, most mycotoxin sequestering agents had the ability to bind to AFB1, but most products, except activated charcoal, failed to sequester DON and ZEA.

## 1. Introduction

Mycotoxins in feeds can have detrimental effects on the growth performance and health of pigs [1,2,3]. Aflatoxin, deoxynivalenol (DON), and zearalenone (ZEA) often occur in the feed ingredients of pigs during the production of plants and the feed production process. Aflatoxin is produced by *Aspergillus* fungi, and DON and ZEA are generated by *Fusarium* fungi. Mycotoxins in feedstuffs have been reported to cause harmful effects on the immune system, ileal amino acid digestibility, feed intake, and body weight gain in pigs [1,2,4], indicating that dietary mycotoxins can cause economic losses in swine production. Meta-analyses on the influence of mycotoxins in swine diets have shown dose-dependent reductions in the feed intake and body weight gain of pigs by dietary aflatoxin, DON, and ZEA [5,6,7].

One of the methods used to overcome the negative effects of mycotoxins in animal feeds is to add toxin sequestering agents to feeds [2]. Adding mycotoxin binders to mycotoxin-contaminated diets improved growth performance and nutrient digestibility in pigs [8,9,10]. However, the effects of toxin sequestering agents are variable, depending on the toxin binders, target mycotoxins, and animal species [2,11]. Animal experiments to determine the efficacy of the mycotoxin sequestering agents are costly and time-consuming. Alternatively, in vitro procedures mimicking the conditions in the stomach and the small intestine of pigs [12] are available for testing mycotoxin sequestering products [13,14,15,16,17,18]. In some experiments [13,14], however, the intestinal environment of animals was not fully simulated. In previous studies [15,17], mycotoxin sequestering agents were tested for aflatoxin and DON in the gastrointestinal environment of pigs. The effectiveness of mycotoxin sequestering agents for aflatoxin has been well documented [13,19,20]. However, their effects on DON and ZEA, which are often co-contaminated in animal feeds, have been unknown or debatable [2,11]. Additionally, information on the mycotoxin sequestering efficacy of the yeast cell wall is very limited. To bridge this gap, we determined the efficacy of 12 mycotoxin sequestering agents for aflatoxin B1 (AFB1), DON, and ZEA using an in vitro method that simulated the gastrointestinal tract of pigs.

## 2. Materials and Methods

### 2.1. Mycotoxin Sequestering Agents and Toxin Preparation

Twelve toxin sequestering agents were tested: seven bentonite products (bentonite A, B, C, D, E, F, and G), two aluminosilicate products (aluminosilicate A and B), a heulandite product, an activated charcoal product, and a yeast cell wall product. Bentonite A, B, and E consisted mainly of calcium montmorillonite, but bentonite C, D, and G consisted mainly of sodium montmorillonite. Bentonite D additionally contained phytogenic substances, enzymes, and microorganisms. Bentonite F contained both calcium and sodium ions with an organic compound. Bentonite G additionally contained sepiolite. Aluminosilicate A consisted of a hydrated sodium silicate and calcium silicate with an organic compound. Aluminosilicate B included an activated sodium and calcium aluminosilicate. The heulandite product, a stable crystal structure with negatively charged ions, was composed of heat-treated clinoptilolite. The activated charcoal product was produced from a coconut shell. The yeast cell wall was composed of organic polysaccharides, including hydrolyzed yeast cell walls and mannan-oligosaccharides.

Standard solutions of AFB1 (2 μg/mL), DON (100 μg/mL), and ZEA (100 μg/mL) in acetonitrile (Romer Labs Diagnostic GmbH, Tulln, Austria) were diluted to 10, 1000, and 250 ng/mL, respectively, using distilled water. The quantification ranges of the enzyme-linked immunosorbent assay (ELISA) kit used for the quantitative analysis of toxins were from 2 to 50 ng/mL for AFB1, 250 to 5000 ng/mL for DON, and 25 to 1000 ng/mL for ZEA.

### 2.2. In Vitro Procedure

The in vitro procedure used in the present work was based on methods mimicking the gastrointestinal tract of pigs [15,21] with minor modifications. In a 50 mL-conical tube containing 2.5 mL of phosphate buffer (0.1 M, pH 6.0), 12.5 mg of a sequestering agent (0.5% suspension) and 5 mL of diluted mycotoxin standard solution were added. The pH was adjusted to approximately 2.0 by adding 300 μL of 1 M HCl to simulate the condition of the stomach. Then, each conical tube was incubated in a shaking incubator at 39 °C for 2 h. After a 2 h incubation, 1 mL of phosphate buffer (0.2 M, pH 6.8) and 300 μL of 1 M NaOH were added to the tube to achieve pH 6.8, which simulated the small intestine. Then, the solution was incubated at 39 °C for 4 h. After the incubation, the mixture was centrifuged at 2800× *g* for 10 min and the supernatant was collected to analyze residual unbound AFB1, DON, and ZEA. The ELISA test kits were used to determine the residual AFB1 (AgraQuant^®^ Aflatoxin B1; COKAQ8000, Romer Labs Inc., Singapore), DON (AgraQuant^®^ Deoxynivalenol; COKAQ4000, Romer Labs Inc., Singapore), and ZEA (AgraQuant^®^ Zearalenone; COKAQ5100, Romer Labs Inc., Singapore) concentrations. The in vitro procedures were performed in triplicate for each sequestering agent.

### 2.3. Calculation and Statistical Analysis

The sequestering rates of AFB1, DON, and ZEA by mycotoxin sequestering agents were calculated using the following equation:

(1) Sequestering rate, % =(initial toxin – residual toxin) ÷ initial toxin ×100

where initial toxin (ng/mL) is the initial concentration of mycotoxin (AFB1, DON, or ZEA) in the digestion conical tube, while residual toxin (ng/mL) is the concentration of mycotoxin (AFB1, DON, or ZEA) unbound by mycotoxin sequestering agent in the conical tube after the digestion procedure. The concentration of unbound AFB1, DON, or ZEA by mycotoxin sequestering agent was calculated in comparison to the control group containing no mycotoxin sequestering agent.

Statistical analyses were performed using the MIXED procedure of SAS (SAS Inst. Inc., Cary, NC, USA). The statistical model included the mycotoxin sequestering agent as a fixed variable and the replication as a random variable. Least squares means were separated using the PDIFF option of the MIXED procedure with Tukey’s adjustment. Statistical significance was determined at a *p*-value less than 0.05.

## 3. Results

### 3.1. Sequestering Rates of AFB1 by Mycotoxin Sequestering Agents

The sequestering rates of AFB1 by bentonite A, bentonite B, bentonite G, aluminosilicate B, and activated charcoal were greater (*p* < 0.05) than those by bentonite D and yeast cell wall (Table 1). All mycotoxin sequestering agents tested were able to bind to AFB1 with a high efficacy (>92%).

### 3.2. Sequestering Rates of DON by Mycotoxin Sequestering Agents

The DON sequestering rate of activated charcoal (99.1%) was greater (*p* < 0.05) than that of other products (Table 2). All the products tested, except activated charcoal, did not effectively sequester DON.

### 3.3. Sequestering Rates of ZEA by Mycotoxin Sequestering Agents

The sequestering rates of ZEA by bentonite F, aluminosilicate A, and activated charcoal were the greatest (*p* < 0.05) among the tested mycotoxin sequestering agents (Table 3). However, all the other products did not effectively sequester ZEA.

## 4. Discussion

Mycotoxins such as AFB1, DON, and ZEA are often present in feed ingredients during the growth and drying of plants in the field or during long-term storage post-harvest [22,23]. Mycotoxins in feedstuffs have negative effects on the productivity of pigs [1,2,4]. One of the strategies used to overcome the detrimental effects of feed mycotoxins is to add a mycotoxin sequestering agent, often called a toxin binder, to diets [10,24]. An in vitro method for testing the efficacy of mycotoxin sequestering agents for AFB1 and DON has been reported previously [15]. The efficacy of a toxin sequestering agent for ZEA is also critical as ZEA and DON, *Fusarium* mycotoxins, are often co-contaminated in animal feeds [25]. In a previous study by Kong et al. [15], five types of sequestering agents were tested, but no products were able to effectively sequester DON. In the present work, therefore, the efficacy of more various sequestering agents for AFB1, DON, and ZEA was determined using an in vitro method that mimicked the intestinal environment of pigs.

The sequestering rates of AFB1 by all mycotoxin sequestering products tested in the present work agree with previous studies [11,15]. Clays consisting of zeolite-clinoptilolite and montmorillonite have been reported to sequester polar groups of AFB1 [26]. Bentonite, an adsorbent clay mostly composed of montmorillonite, has a high affinity to AFB1 due to the polar characteristics of bentonite and AFB1 [27]. Similarly to the present observations, high sequestering rates of bentonite products for AFB1 have been reported in quite a few studies [13,19,20]. The great sequestering ability of two aluminosilicate products in the present work is likely due to the fact that aluminosilicates can form a stable complex with AFB1 at 37 °C [28]. In agreement with the present work, an aluminosilicate product has been shown to effectively bind to AFB1 [17,18]. Heulandite is categorized as a zeolite consisting of tectosilicates and has AFB1 sequestering ability [29], which supports the present results. Activated charcoal is a very porous powder produced by the pyrolysis of organic matter and has been reported to adsorb multiple mycotoxins, including AFB1 [30,31,32]. However, activated charcoal is a nonspecific binder that potentially adsorbs essential nutrients [2]. Yeast cell wall is an organic compound consisting of polysaccharides such as β-D-glucans and mannans that can adsorb AFB1 by hydrogen bonding [33,34], which agrees with the present results.

The relatively low sequestering rates of DON by bentonite products are in agreement with previous reports [15,16,18], indicating that bentonite products do not effectively bind to or digest DON. The lower effectiveness of bentonites for sequestering DON compared with AFB1 may be associated with the lower polarity of DON [2,11]. By the same token, aluminosilicates were not able to bind DON [28] due to the lack of positive cations in the negatively charged aluminosilicate framework [11]. The negative values for the sequestering rate of DON in the present study are likely due to experimental error, analytical error, or both, as also reported in previous work [15]. The activated charcoal product tested in the present work sequestered DON effectively, whereas an activated charcoal product used in a previous work did not sequester DON [15]. Although the reason for this discrepancy is unclear, the sequestering ability of activated charcoal is dependent on physiochemical factors such as the pore size and surface area of the charcoal products [35]. The surface area of commercial activated charcoals varies from 500 m^2^/g to 3500 m^2^/g, depending on the structure materials [36]. The sequestering rate of DON by the yeast cell wall tested in the current study was less than that of a yeast cell wall product used in a previous work [15], which is likely due to its different cellular components that potentially interact with DON [33,37]. However, both yeast cell wall products in this work and the previous work [15] had a less than 25% sequestering rate for DON.

Among the bentonite products, only bentonite F effectively sequestered ZEA in this work. This product has been claimed to have a high affinity to ZEA once hydrated to a polar molecule. As one of the major components of bentonite, montmorillonite in a chemically modified form has been reported to bind ZEA [38]. However, more information on the bentonite products in this work is not available. In the present work, ZEA was well sequestered by aluminosilicate A but not by aluminosilicate B. Aluminosilicates composed of silicates, aluminates, and some interchangeable ions [39] have been reported to not be able to sequester ZEA effectively [2,28]. In contrast, an aluminosilicate product has been shown to sequester ZEA effectively [18], which agrees with the present results obtained for aluminosilicate A. The aluminosilicate A tested in the present work may have been chemically modified to achieve a higher affinity to ZEA, but the specific production procedures are unknown. Similarly to AFB1 and DON, activated charcoal sequestered ZEA effectively as a nonspecific binder [30,31]. The activated charcoal product used in the present work likely has sufficient surface area and pore size for adsorbing ZEA. Factors affecting the effectiveness of activated charcoal include the dose and pH of the product in addition to the pore size and surface area, as mentioned previously [36]. The low sequestering rate of the yeast cell wall product is likely due to the proportional differences in the active components. In a previous in vitro study [27], yeast cell wall products with different components showed variable effects on ZEA adsorption. Yiannikouris et al. [40] suggested that the ZEA binding efficacy of yeast cell wall products was dependent upon the pH conditions. The negative values for the sequestering rate of ZEA are likely due to experimental error, analytical error, or both, similarly to DON.

## 5. Conclusions

In conclusion, most mycotoxin sequestering agents used in the present work were able to bind aflatoxin B1; however, their effects on deoxynivalenol and zearalenone varied based on the in vitro procedure used to mimic the gastrointestinal tract of pigs. Only activated charcoal effectively sequestered deoxynivalenol, and a bentonite product, an aluminosilicate product, and activated charcoal effectively sequestered zearalenone.

## Figures and Tables

**Table 1:** Sequestering rates of aflatoxin B1 by mycotoxin sequestering agents (*n* = 3).

Sequestering Agent	Aflatoxin B1, ng/mL		Aflatoxin B1 Sequestering Rate, %
	Initial	Residual ^1^	
Bentonite A		0.33 ^bcd^	96.7 ^abc^
Bentonite B		0.34 ^bcd^	96.6 ^abc^
Bentonite C		0.42 ^abcd^	95.8 ^abcd^
Bentonite D		0.79 ^a^	92.1 ^d^
Bentonite E		0.47 ^abc^	95.3 ^bcd^
Bentonite F		0.72 ^ab^	92.8 ^cd^
Bentonite G	10.00	0.17 ^cd^	98.3 ^ab^
Aluminosilicate A		0.50 ^abd^	95.0 ^bcd^
Aluminosilicate B		0.03 ^d^	99.7 ^a^
Heulandite		0.41 ^abcd^	95.9 ^abcd^
Activated charcoal		0.15 ^cd^	98.5 ^ab^
Yeast cell wall		0.77 ^a^	92.3 ^d^
SEM ^2^		0.08	0.77
*p*-value		<0.001	<0.001

^a–d^ Values within a column without a common superscript letter differ (*p* < 0.05). ^1^ The concentration of unbound aflatoxin B1 by mycotoxin sequestering agent was calculated in comparison to the control group containing no mycotoxin sequestering agent. ^2^ SEM = standard error of the means.

**Table 2:** Sequestering rates of deoxynivalenol by mycotoxin sequestering agents (*n* = 3).

Sequestering Agent	Deoxynivalenol, ng/mL		Deoxynivalenol Sequestering Rate, %
	Initial	Residual ^1^	
Bentonite A		910 ^ab^	9.0 ^bc^
Bentonite B		836 ^ab^	16.4 ^bc^
Bentonite C		1067 ^a^	−6.7 ^c^
Bentonite D		661 ^b^	33.9 ^b^
Bentonite E		1020 ^a^	−2.0 ^c^
Bentonite F		911 ^ab^	8.9 ^bc^
Bentonite G	1000	947 ^ab^	5.3 ^bc^
Aluminosilicate A		918 ^ab^	8.2 ^bc^
Aluminosilicate B		996 ^a^	0.4 ^c^
Heulandite		1051 ^a^	−5.1 ^c^
Activated charcoal		9 ^c^	99.1 ^a^
Yeast cell wall		990 ^a^	1.0 ^c^
SEM ^2^		58.4	5.84
*p*-value		<0.001	<0.001

^a*–*c^ Values within a column without a common superscript letter differ (*p* < 0.05). ^1^ The concentration of unbound deoxynivalenol by mycotoxin sequestering agent was calculated in comparison to the control group containing no mycotoxin sequestering agent. ^2^ SEM = standard error of the means.

**Table 3:** Sequestering rates of zearalenone by mycotoxin sequestering agents (*n* = 3).

Sequestering Agent	Zearalenone, ng/mL		Zearalenone Sequestering Rate, %
	Initial	Residual ^1^	
Bentonite A		196.9 ^b^	21.2 ^b^
Bentonite B		255.9 ^a^	−2.3 ^c^
Bentonite C		242.1 ^a^	3.2 ^c^
Bentonite D		272.8 ^a^	−9.1 ^c^
Bentonite E		270.9 ^a^	−8.4 ^c^
Bentonite F		7.4 ^c^	97.0 ^a^
Bentonite G	250.0	240.9 ^a^	3.6 ^c^
Aluminosilicate A		1.0 ^c^	99.6 ^a^
Aluminosilicate B		256.3 ^a^	−2.5 ^c^
Heulandite		254.1 ^a^	−1.6 ^c^
Activated charcoal		0.0 ^c^	100.0 ^a^
Yeast cell wall		234.9 ^ab^	6.0 ^bc^
SEM ^2^		8.55	3.42
*p*-value		<0.001	<0.001

^a*–*c^ Values within a column without a common superscript letter differ (*p* < 0.05). ^1^ The concentration of unbound zearalenone by mycotoxin sequestering agent was calculated in comparison to the control group containing no mycotoxin sequestering agent. ^2^ SEM = standard error of the means.

## Data Availability

The data presented in the current work are available.
